# Supplementary material for: New Terpendole Congeners, Inhibitors of Sterol O-Acyltransferase, Produced by Volutella citrinella BF-0440
Source: Molecules. 2020 Jul 6;25(13):3079. doi: 10.3390/molecules25133079 (PMC7411735; doi:10.3390/molecules25133079)
Supplement: Supplementary file 1 [file molecules-25-03079-s001.pdf]

molecules

MDPI

Article

New terpendole congeners, inhibitors of sterol *O*-acyltransferase, produced by *Volutella citrinella* BF-0440

Elyza Aiman Azizah Nur[1], Keisuke Kobayashi[1,2], Ai Amagai[3], Taichi Ohshiro[1,3,4], and Hiroshi Tomoda[1,3]*

[1] Department of Microbial Chemistry, Graduate School of Pharmaceutical Sciences, Kitasato University, Tokyo, Japan; ml18124@st.kitasato-u.ac.jp (E.A.A.N); kobayashikei@pharm.kitasato-u.ac.jp (K.K)
[2] Medicinal Research Laboratories, School of Pharmacy, Kitasato University, Tokyo, Japan
[3] Department of Microbial Chemistry, School of Pharmacy, Kitasato University, Tokyo, Japan; pp14007@st.kitasato-u.ac.jp (A.A)
[4] Present address, ITOCHU Collaborative Research-Molecular Targeted Cancer Treatment for Next Generation, Graduate School of Medicine, Nagoya University, Aichi, Japan; tohshiro@med.nagoya-u.ac.jp (T.O)
* Correspondence: tomodah@pharm.kitasato-u.ac.jp (H.T)

List of Figures

Figure S1: ^{1}H-NMR spectrum of terpendole O (**2**) in DMSO- d_6.
Figure S2: ^{13}C-NMR spectrum of terpendole O (**2**) in DMSO- d_6.
Figure S3: HSQC spectrum of terpendole O (**2**) in DMSO- d_6.
Figure S4: ^{1}H-^{1}H COSY spectrum of terpendole O (**2**) in DMSO- d_6.
Figure S5: HMBC spectrum of terpendole O (**2**) in DMSO- d_6.
Figure S6: NOESY spectrum of terpendole O (**2**) in DMSO-d_6.
Figure S7: ^{1}H-NMR spectrum of terpendole O (**2**) in $CDCl_3$.
Figure S8: ^{13}C-NMR spectrum of terpendole O (**2**) in $CDCl_3$.
Figure S9: HSQC spectrum of terpendole O (**2**) in $CDCl_3$.
Figure S10: ^{1}H-^{1}H COSY spectrum of terpendole O (**2**) in $CDCl_3$.
Figure S11: HMBC spectrum of terpendole O (**2**) in $CDCl_3$.
Figure S12: NOESY spectrum of terpendole O (**2**) in $CDCl_3$.
Figure S13: ^{1}H-NMR spectrum of terpendole N (**1**) in DMSO- d_6.
Figure S14: ^{13}C-NMR spectrum of terpendole N (**1**) in DMSO- d_6.
Figure S15: HSQC spectrum of terpendole N (**1**) in DMSO- d_6.
Figure S16: ^{1}H-^{1}H COSY spectrum of terpendole N (**1**) in DMSO- d_6.
Figure S17: HMBC spectrum of terpendole N (**1**) in DMSO- d_6.
Figure S18: ROESY spectrum of terpendole N (**1**) in DMSO- d_6.
Figure S19: ^{1}H-NMR spectrum of terpendole P (**3**) in DMSO- d_6.
Figure S20: ^{13}C-NMR spectrum of terpendole P (**3**) in DMSO- d_6.
Figure S21: HSQC spectrum of terpendole P (**3**) in DMSO- d_6.
Figure S22: ^{1}H-^{1}H COSY spectrum of terpendole P (**3**) in DMSO- d_6.
Figure S23: HMBC spectrum of terpendole P (**3**) in DMSO- d_6.
Figure S24: ROESY spectrum of terpendole P (**3**) in DMSO- d_6.
Table S1: ^{1}H and ^{13}C NMR chemical shifts of terpendole O (**2**) in $CDCl_3$.

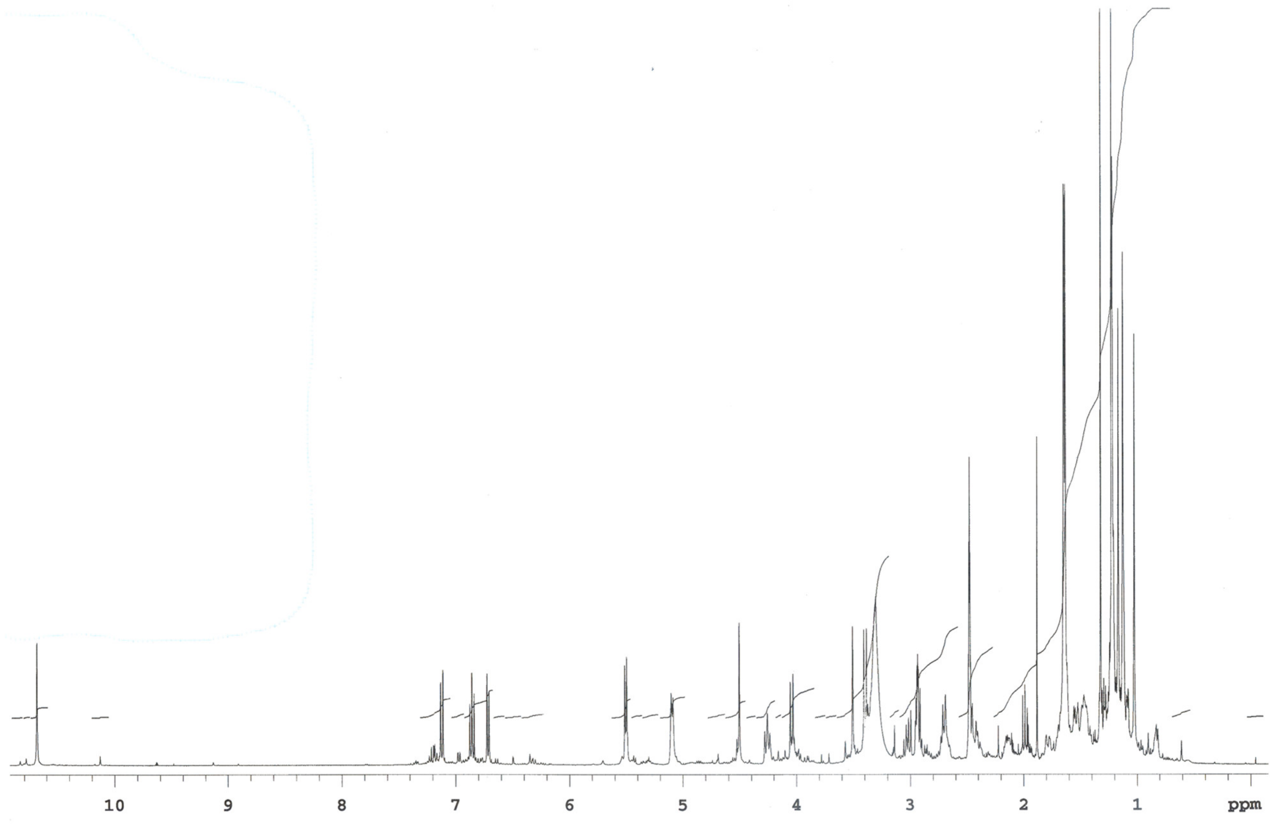

Figure S1. ^{1}H-NMR spectrum of terpendole O (**2**) in DMSO- d_6.

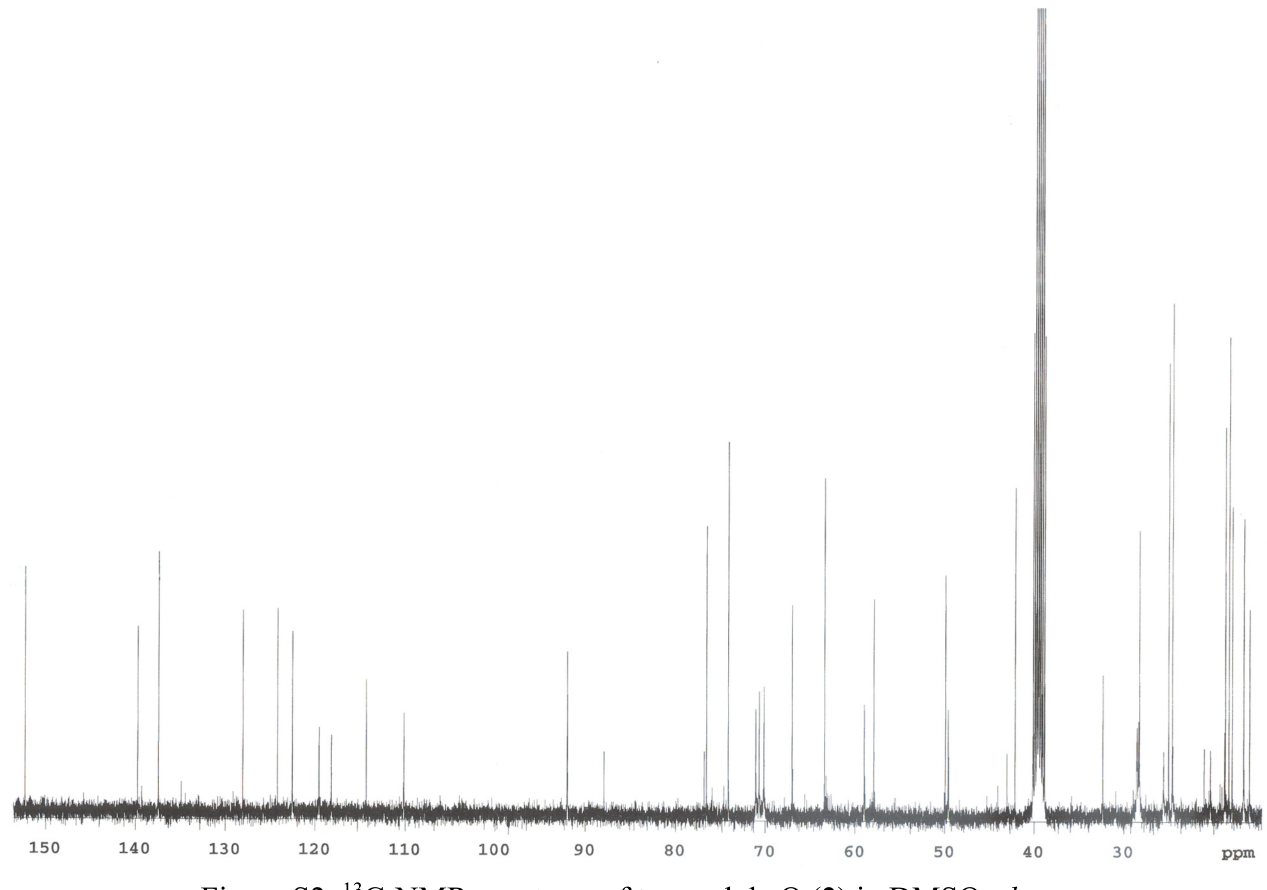

Figure S2. ^{13}C-NMR spectrum of terpendole O (**2**) in DMSO- d_6.

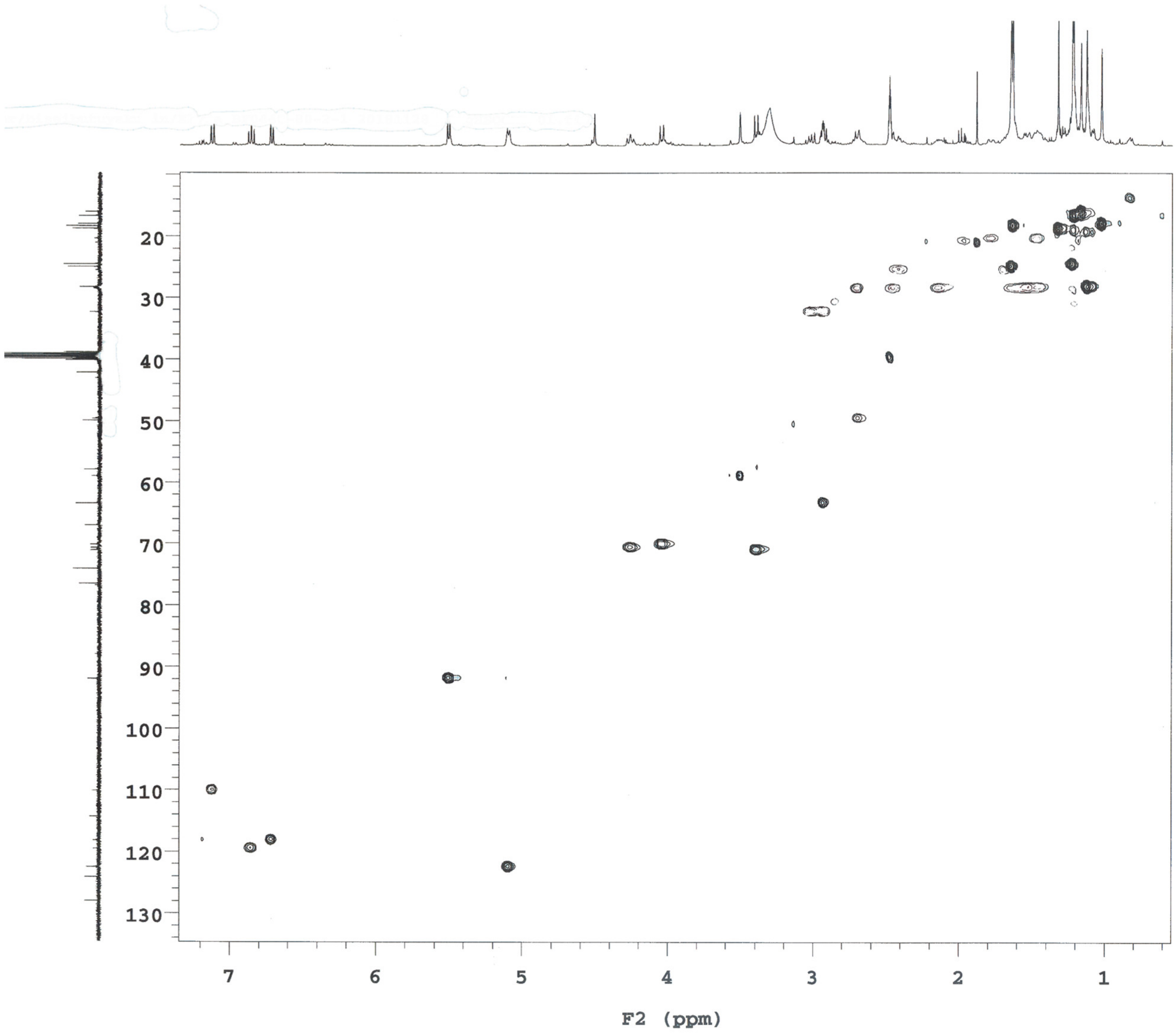

Figure S3. HSQC spectrum of terpendole O (**2**) in DMSO- d_6.

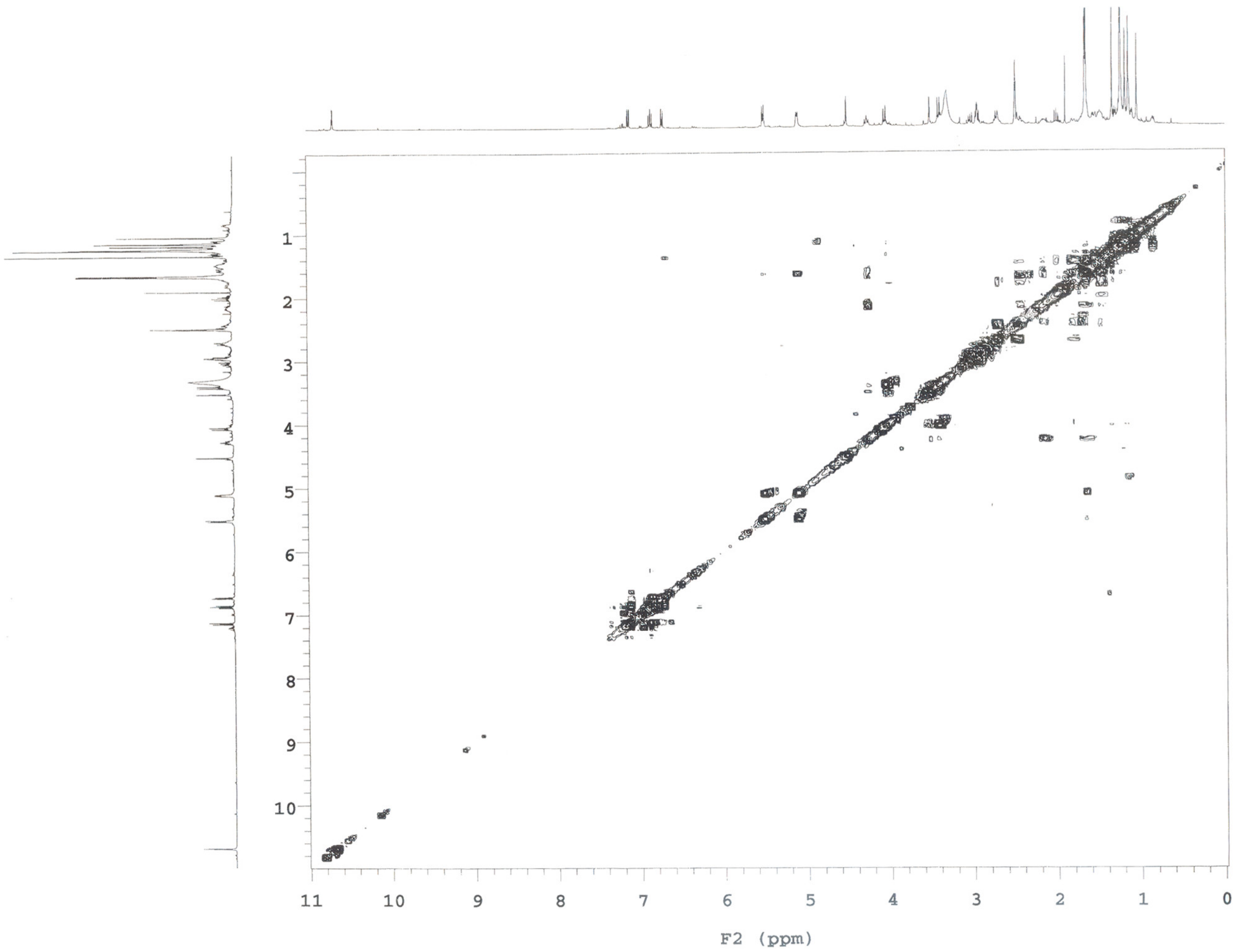

Figure S4. ^{1}H-^{1}H COSY spectrum of terpendole O (**2**) in DMSO- d_6.

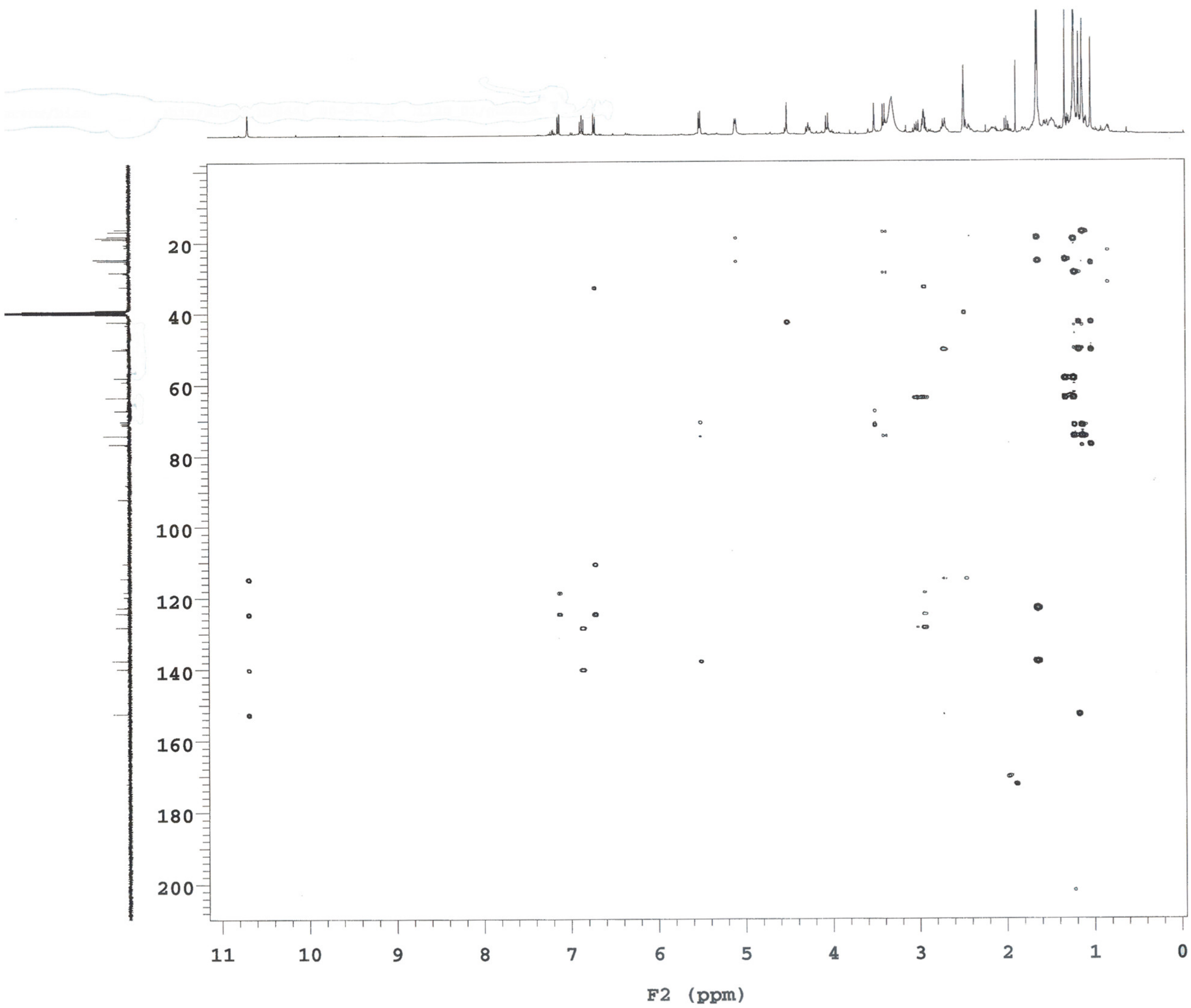

Figure S5. HMBC spectrum of terpendole O (**2**) in DMSO- d_6.

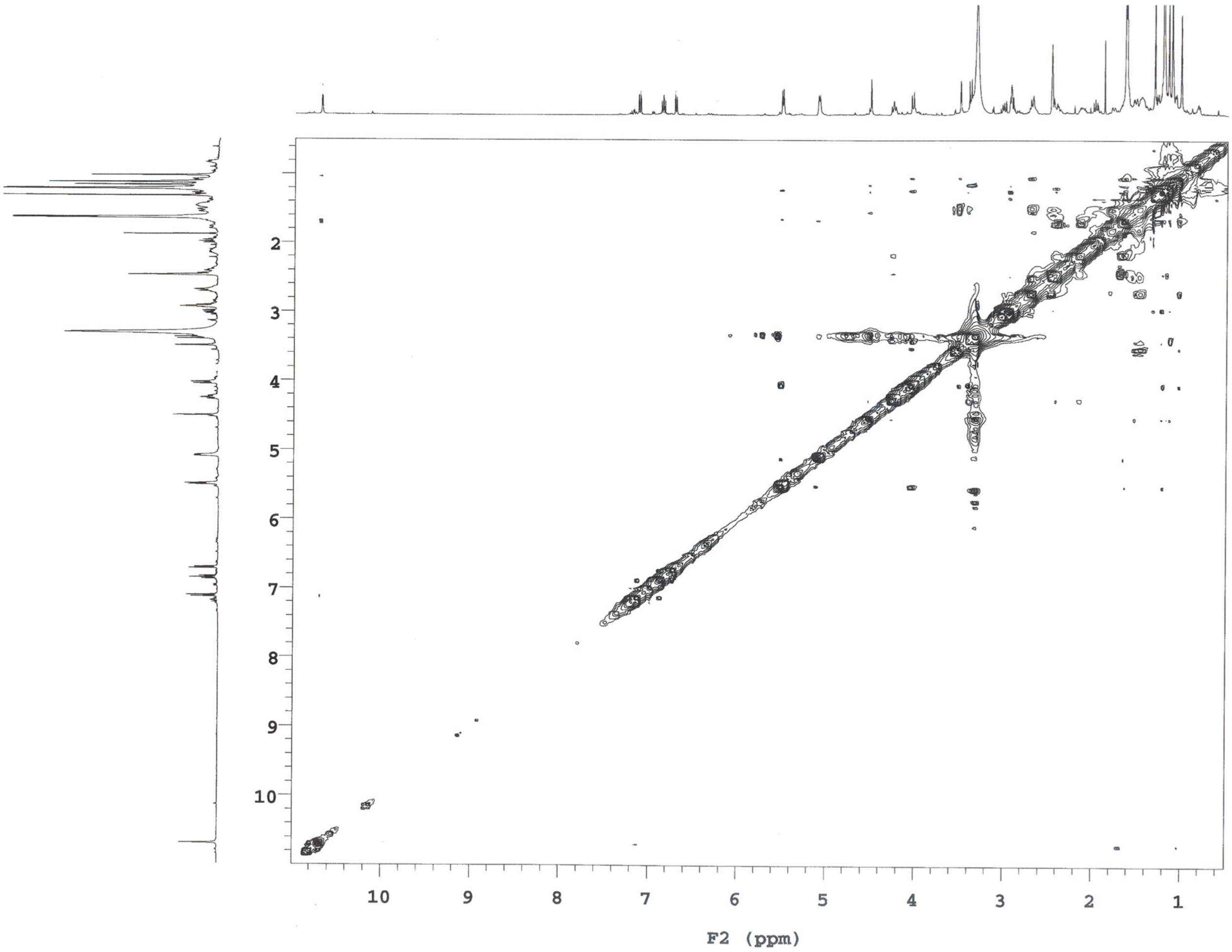

Figure S6. NOESY spectrum of terpendole O (**2**) in DMSO-d_6.

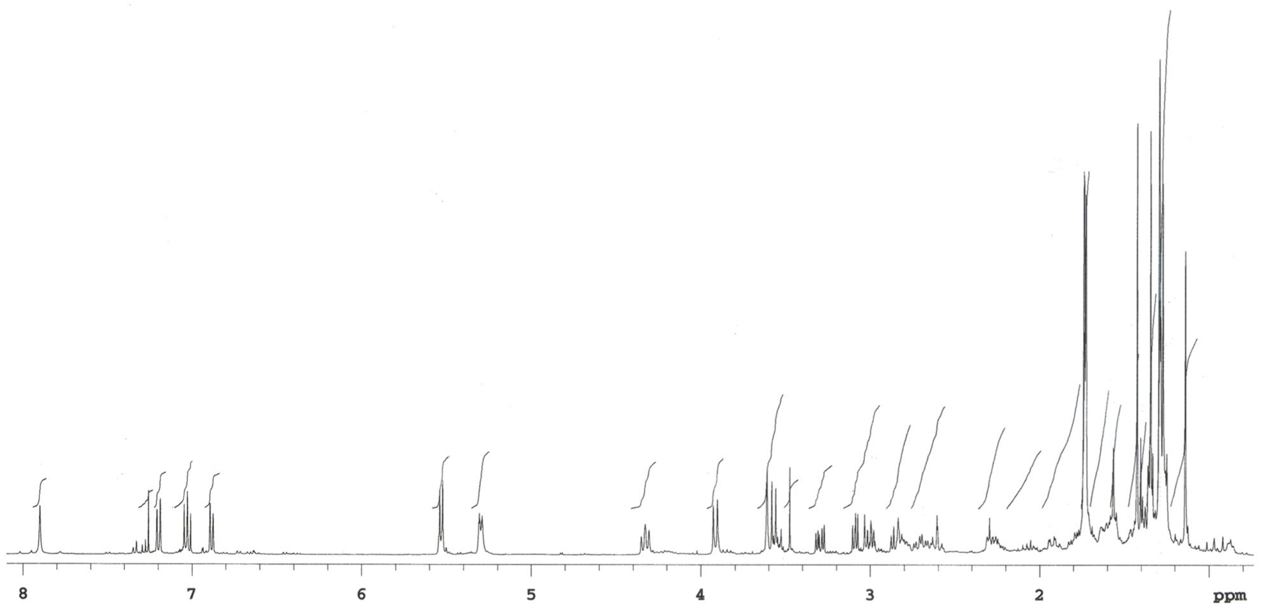

Figure S7. ^{1}H-NMR spectrum of terpendole O (**2**) in $CDCl_3$.

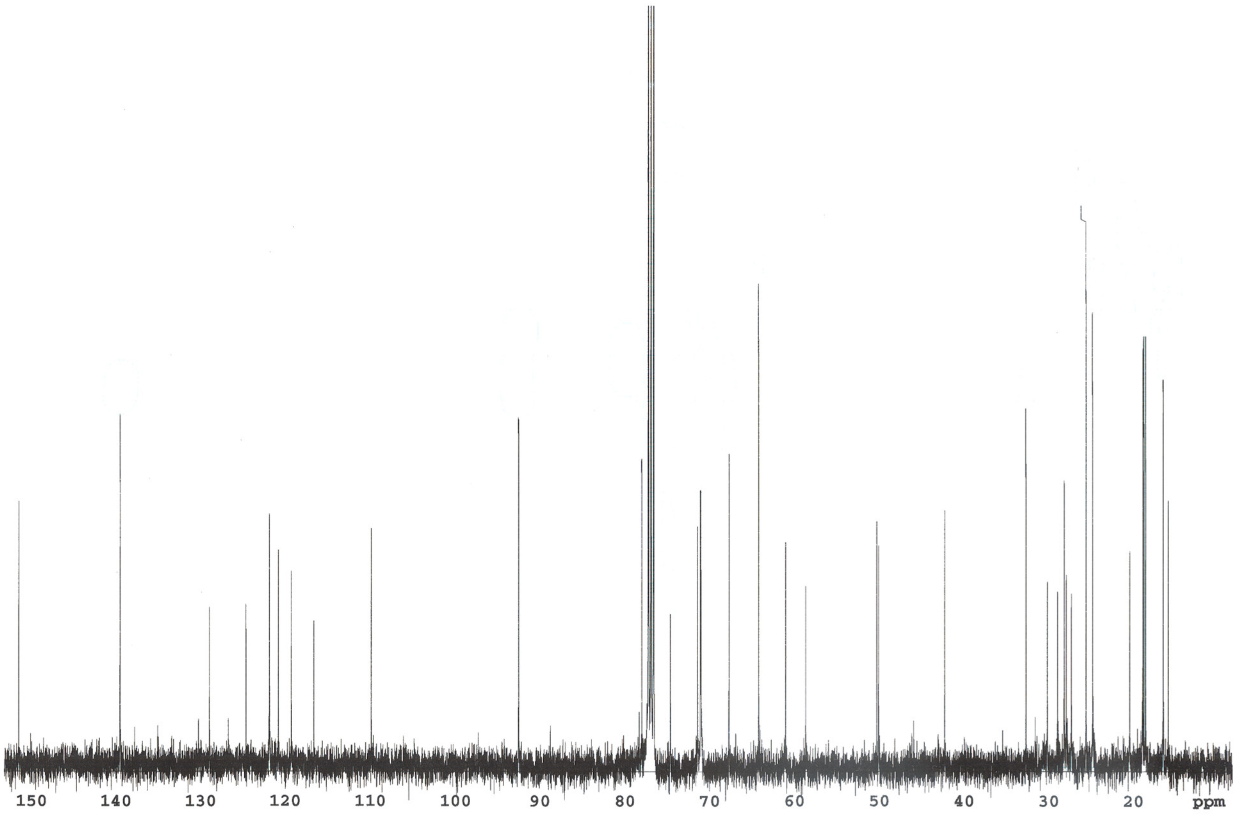

Figure S8. ^{13}C-NMR spectrum of terpendole O (**2**) in $CDCl_3$.

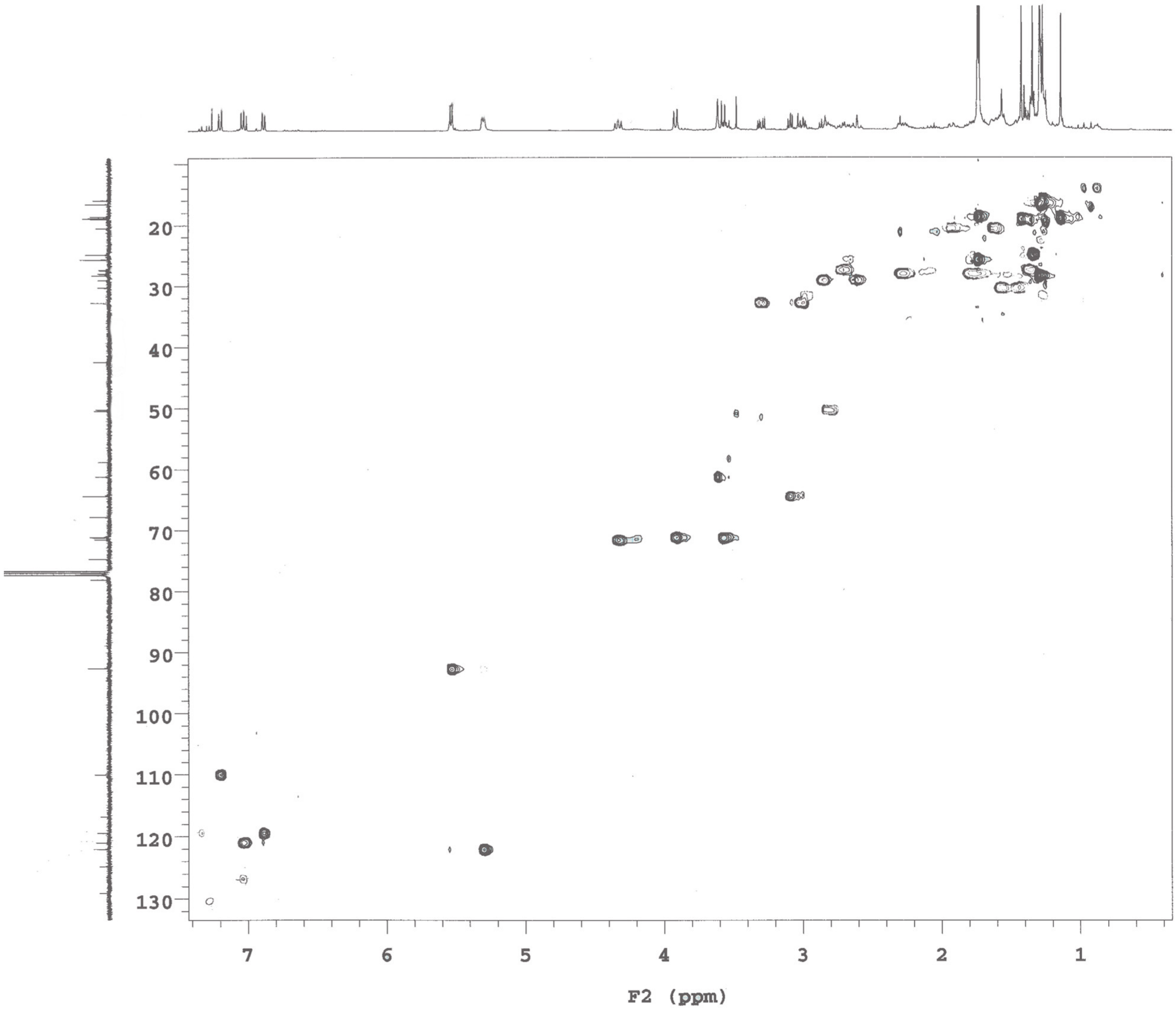

Figure S9. HSQC spectrum of terpendole O (**2**) in $CDCl_3$.

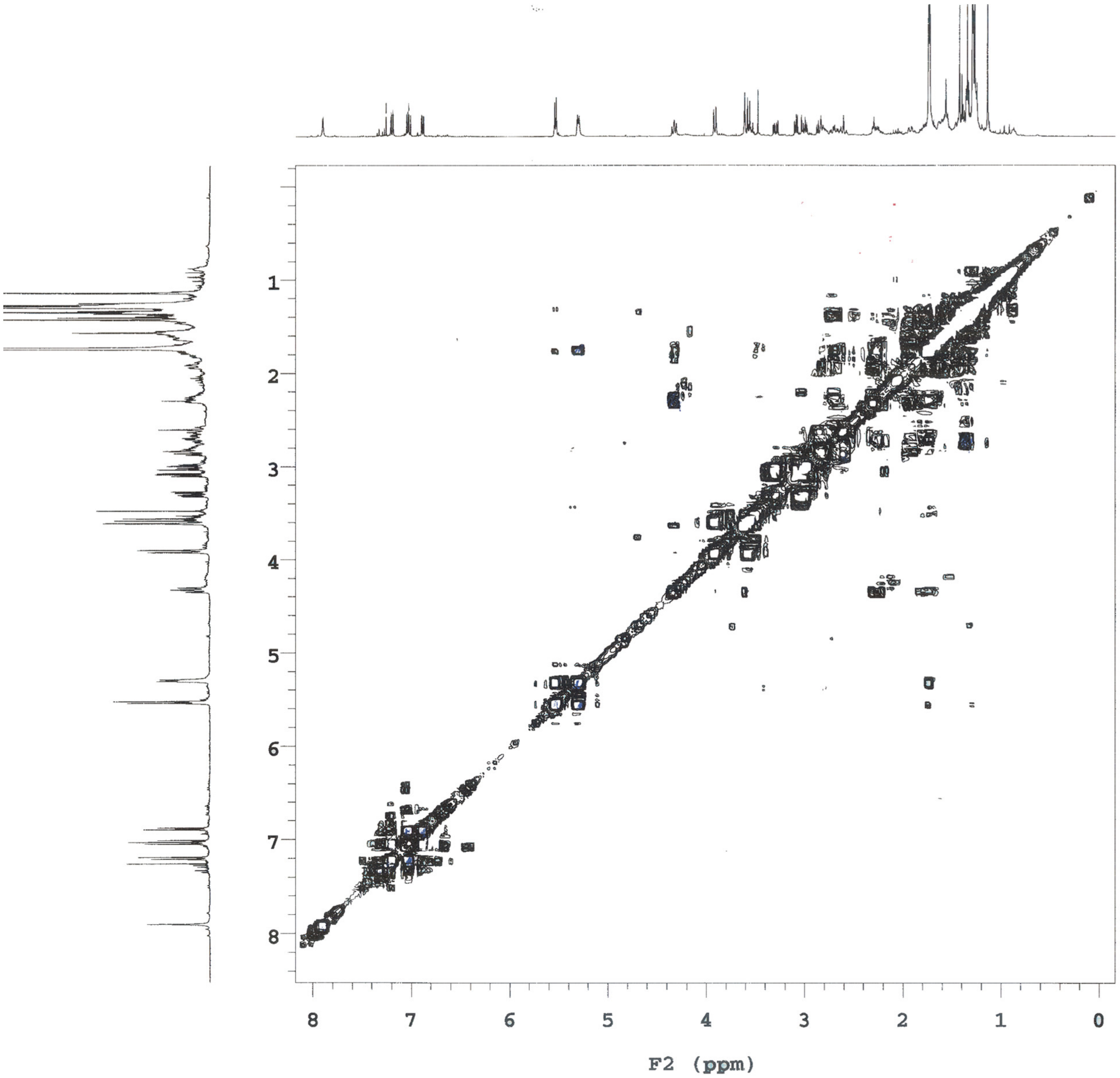

Figure S10. ^{1}H-^{1}H COSY spectrum of terpendole O (**2**) in $CDCl_3$.

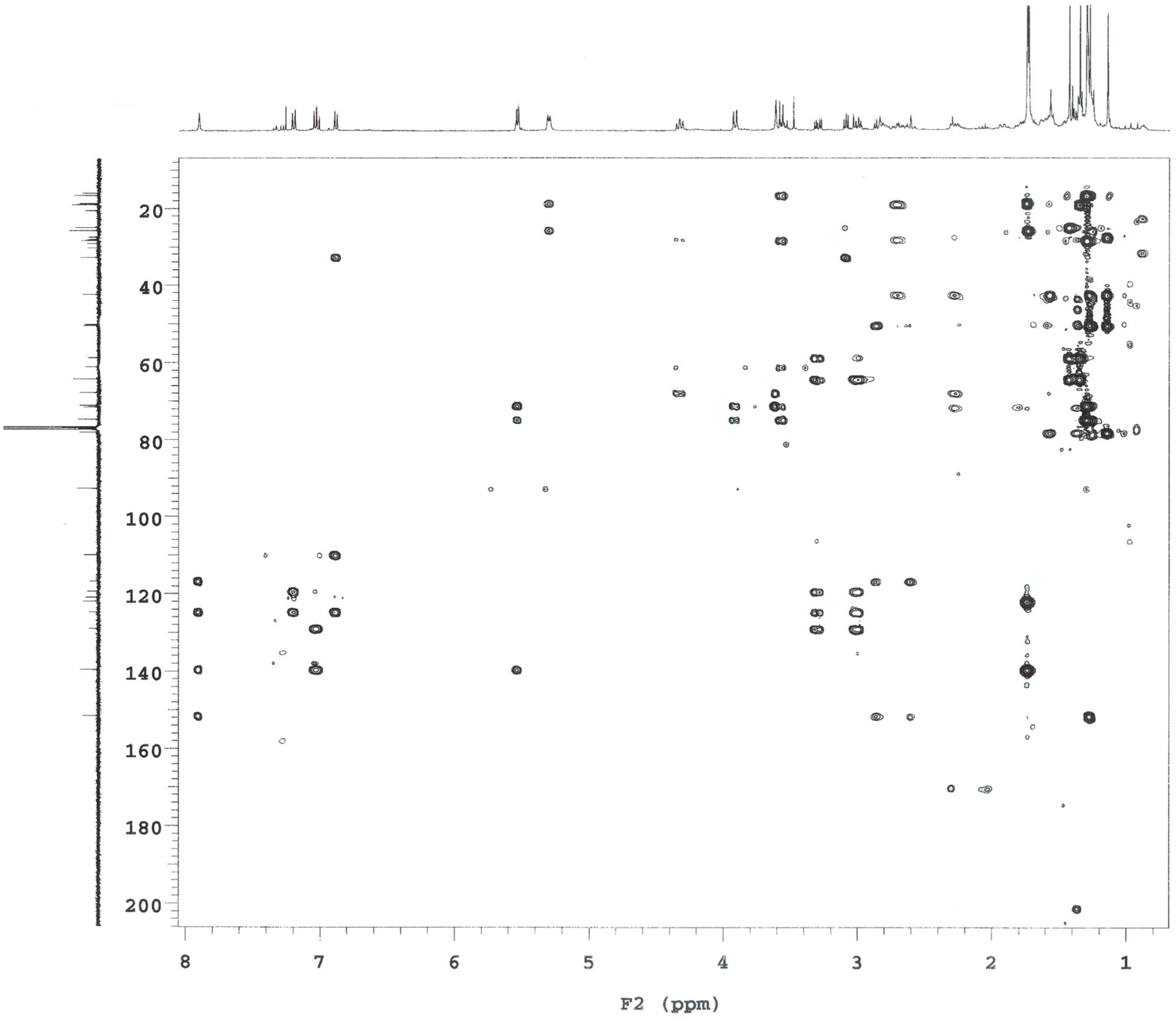

Figure S11. HMBC spectrum of terpendole O (**2**) in $CDCl_3$.

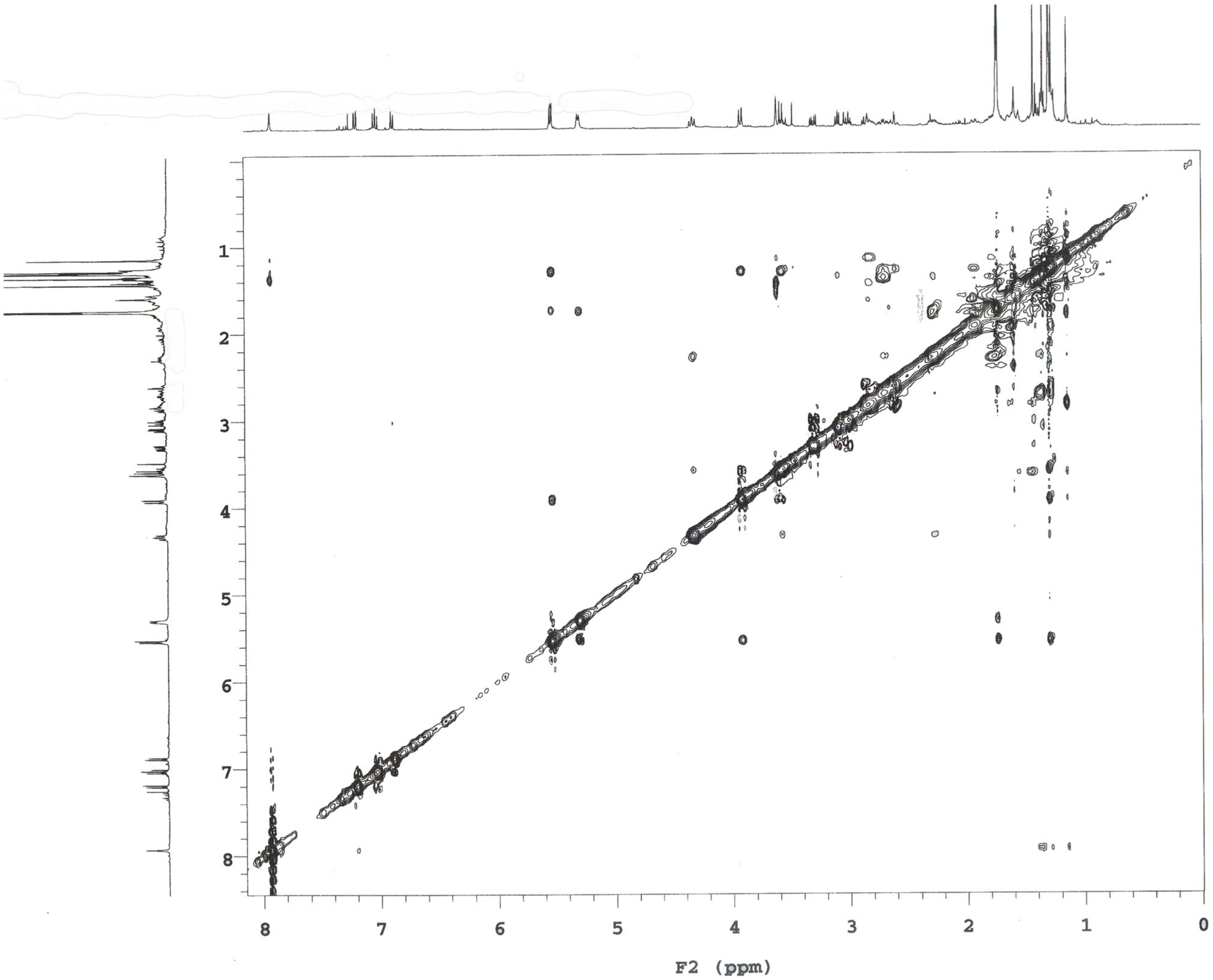

Figure S12. NOESY spectrum of terpendole O (**2**) in $CDCl_3$.

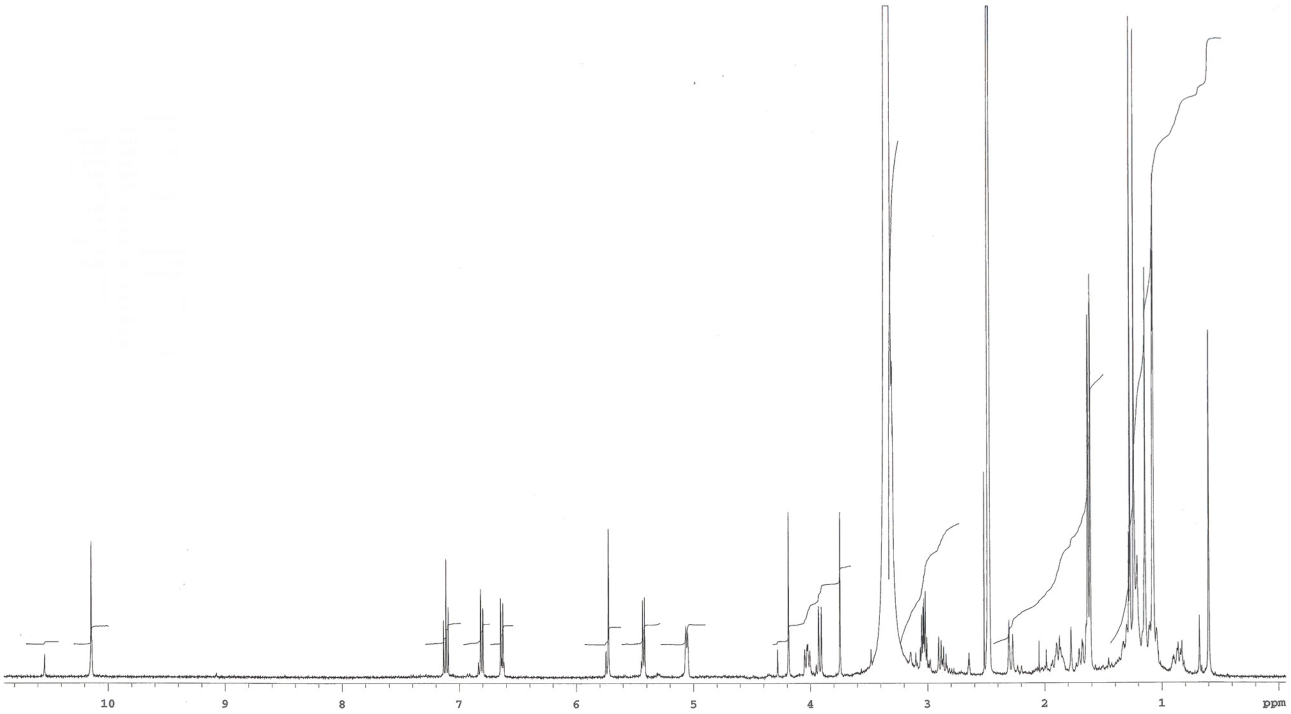

Figure S13. ^{1}H-NMR spectrum of terpendole N (**1**) in DMSO- d_6.

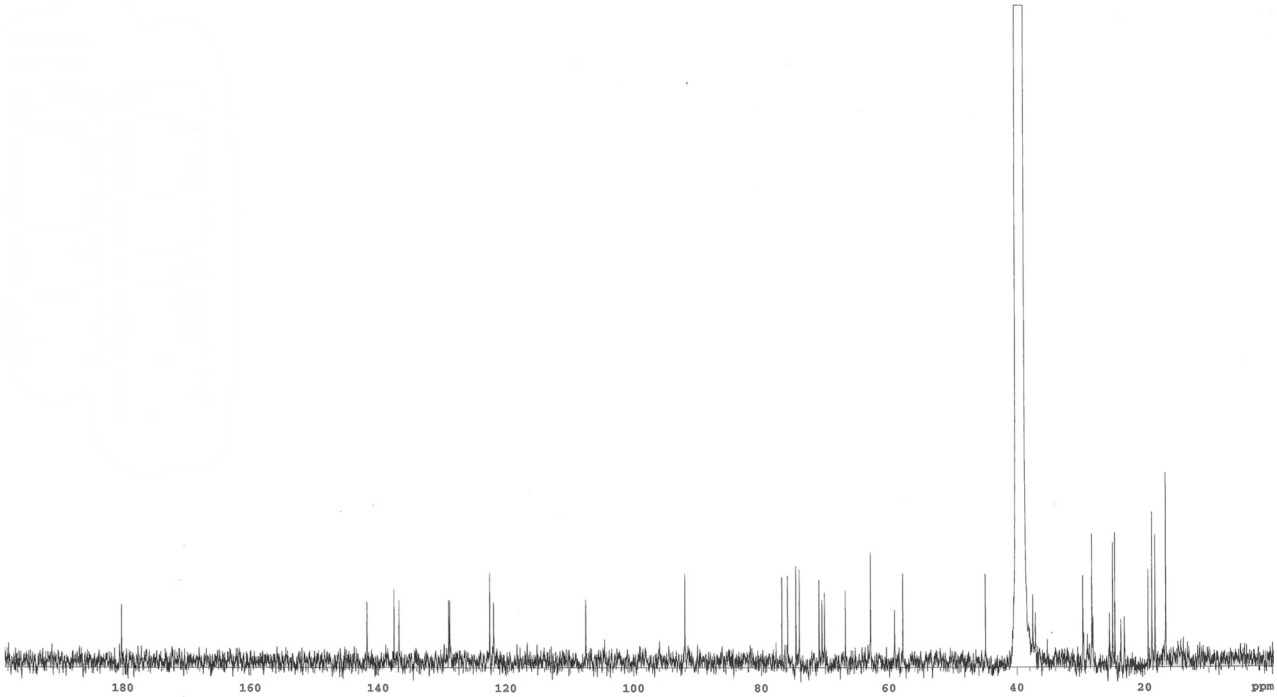

Figure S14. ^{13}C-NMR spectrum of terpendole N (**1**) in DMSO- d_6.

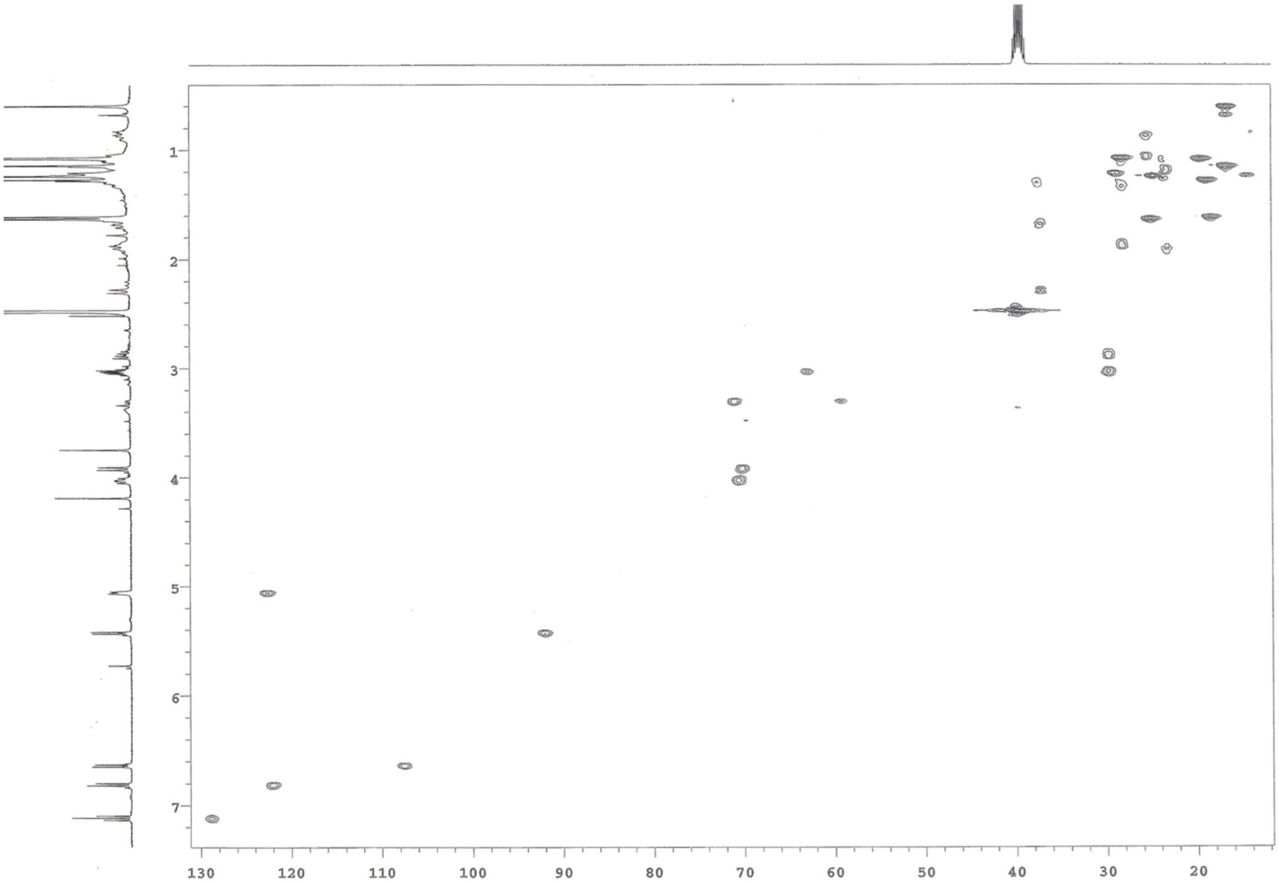

Figure S15. HSQC spectrum of terpendole N (**1**) in DMSO- *d*$_6$.

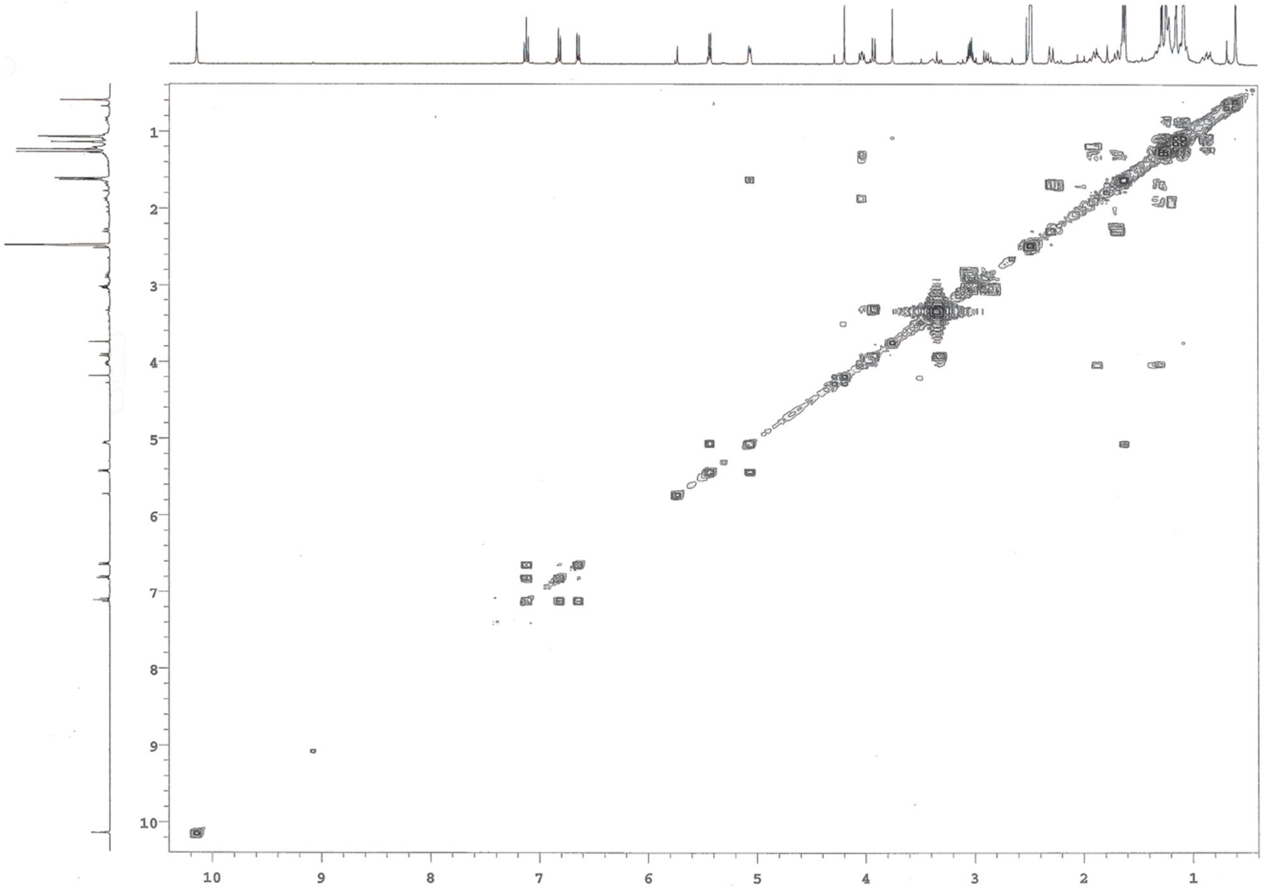

Figure S16. ^{1}H-^{1}H COSY spectrum of terpendole N (**1**) in DMSO- d_6.

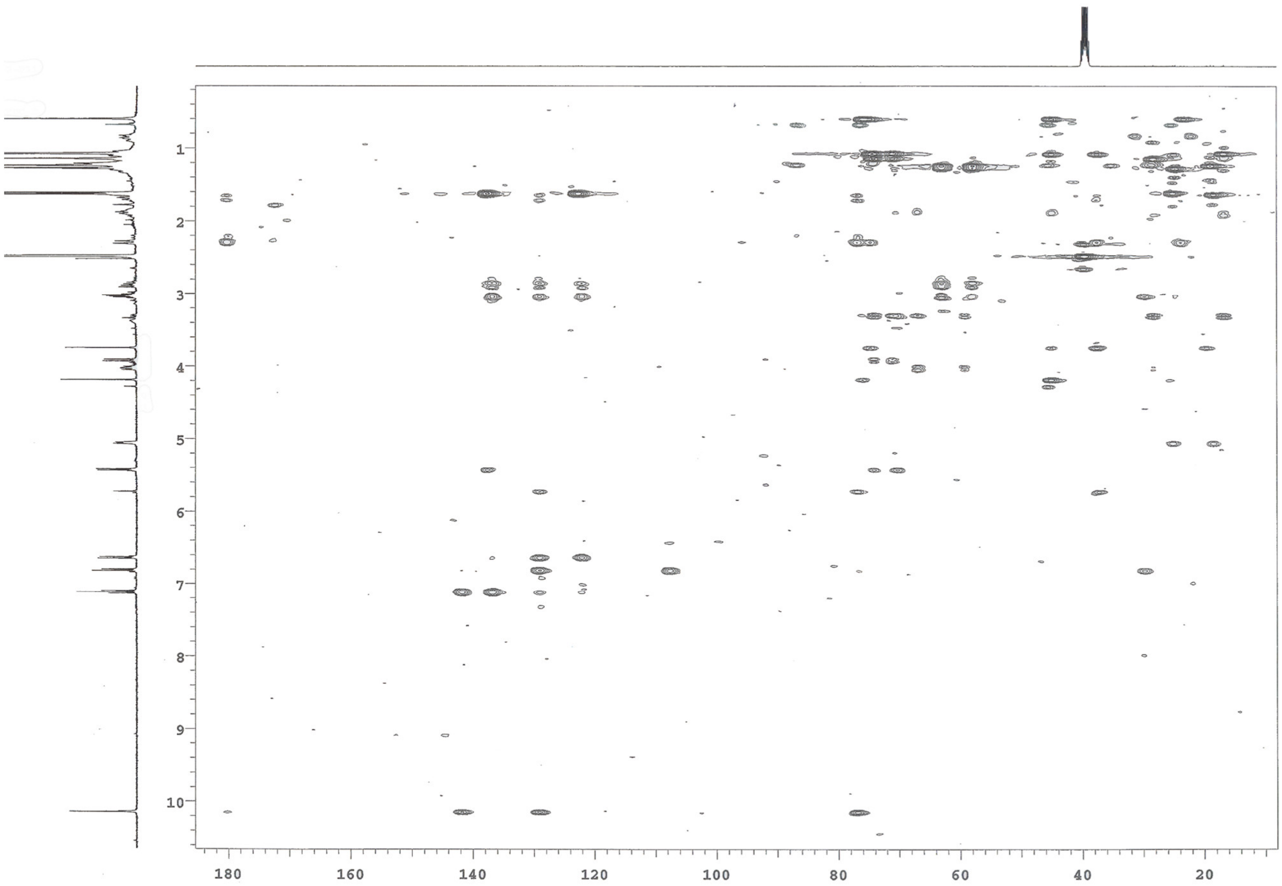

Figure S17. HMBC spectrum of terpendole N (**1**) in DMSO- d_6.

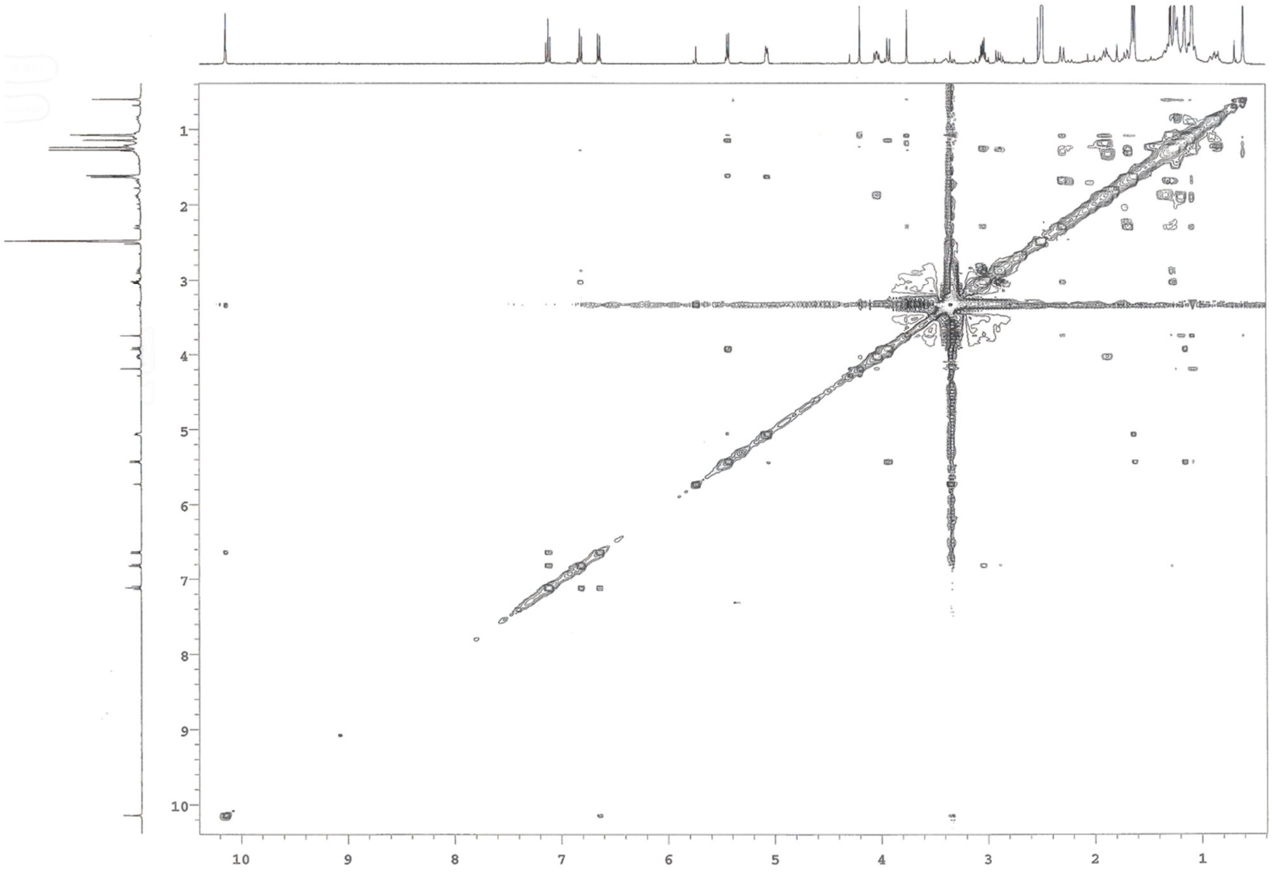

Figure S18. ROESY spectrum of terpendole N (**1**) in DMSO- d_6.

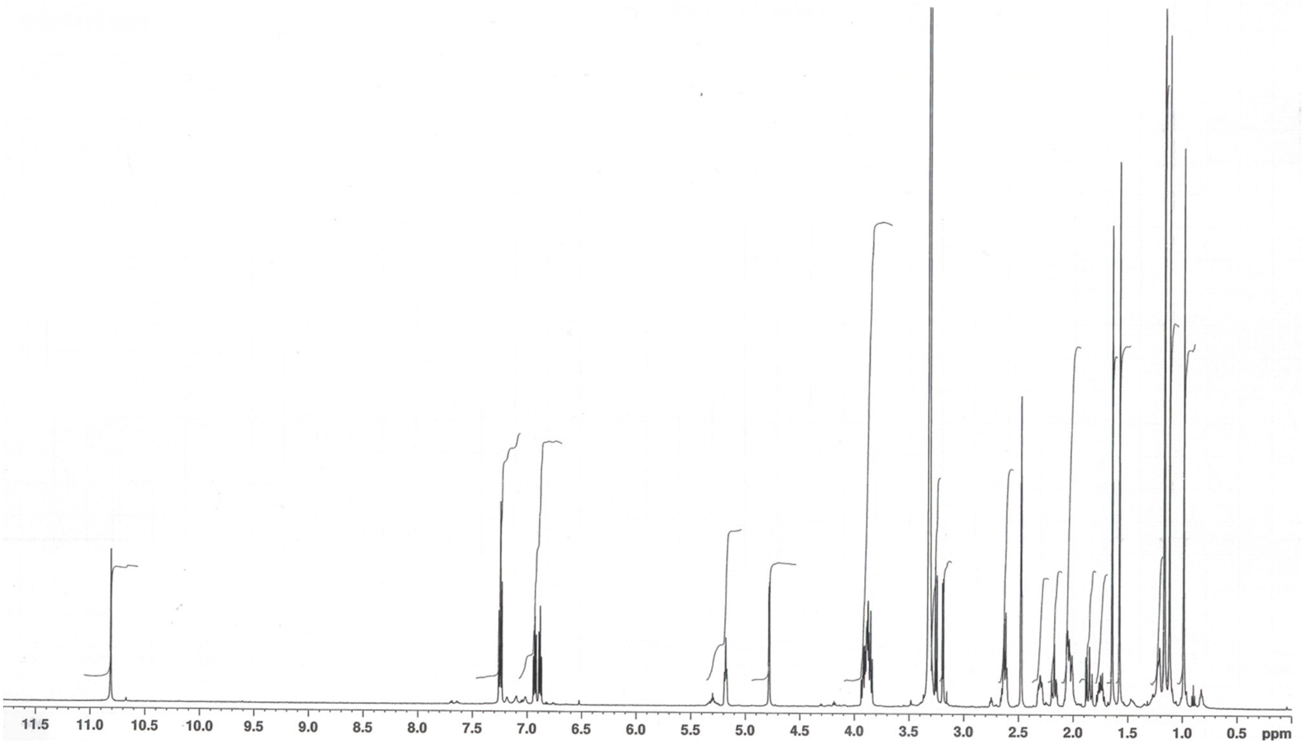

Figure S19. ^{1}H-NMR spectrum of terpendole P (**3**) in DMSO- d_6.

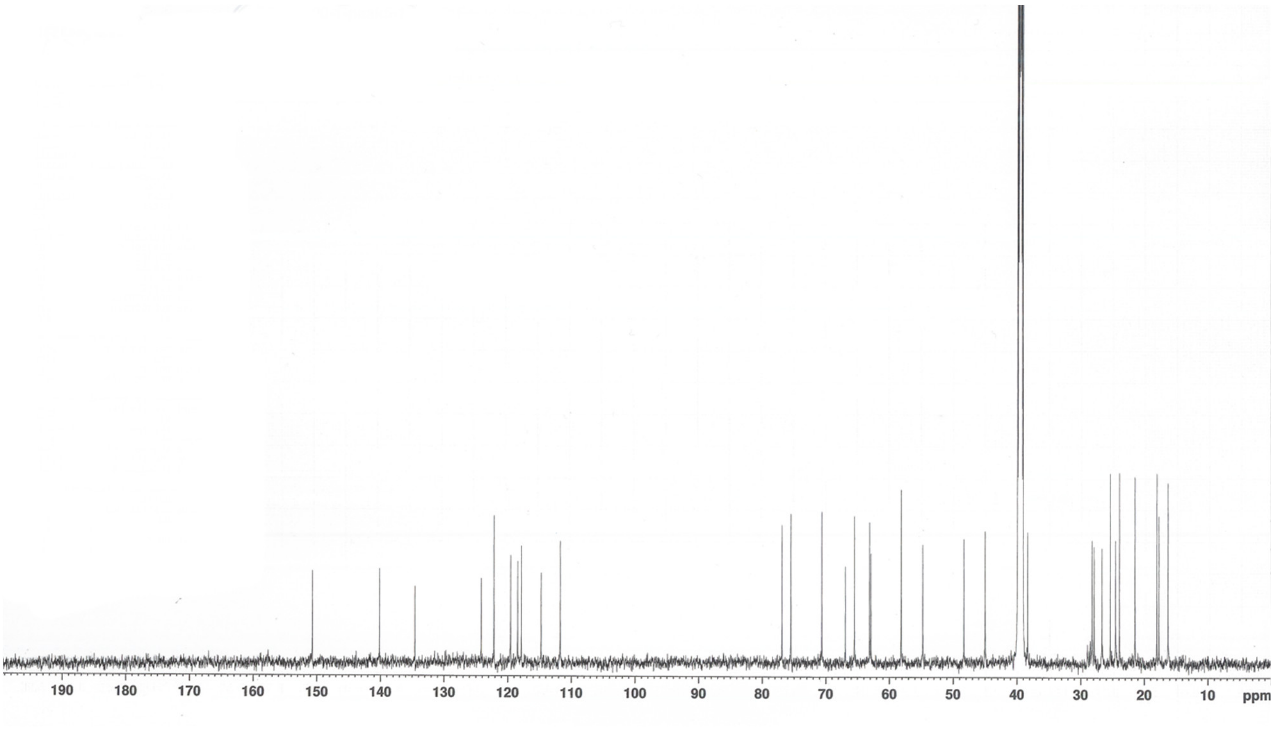

Figure S20. ^{13}C-NMR spectrum of terpendole P (**3**) in DMSO- d_6.

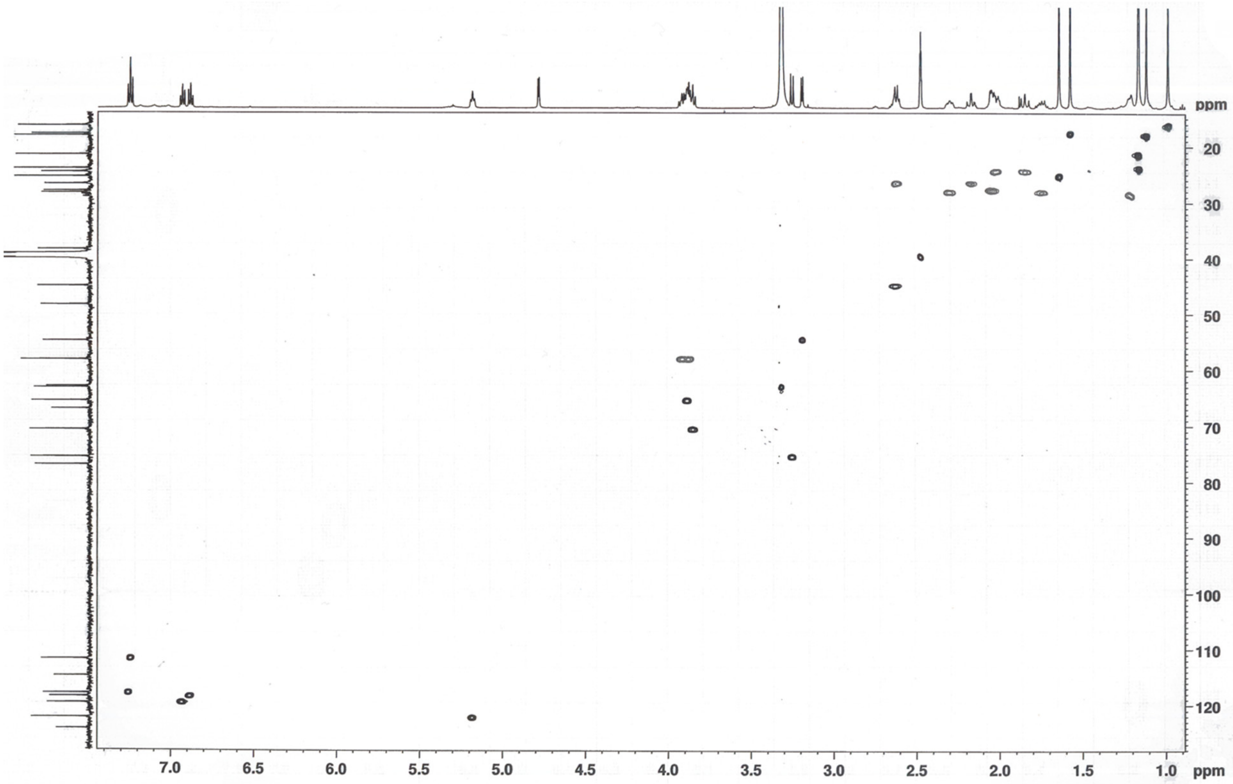

Figure S21. HSQC spectrum of terpendole P (**3**) in DMSO- *d*6.

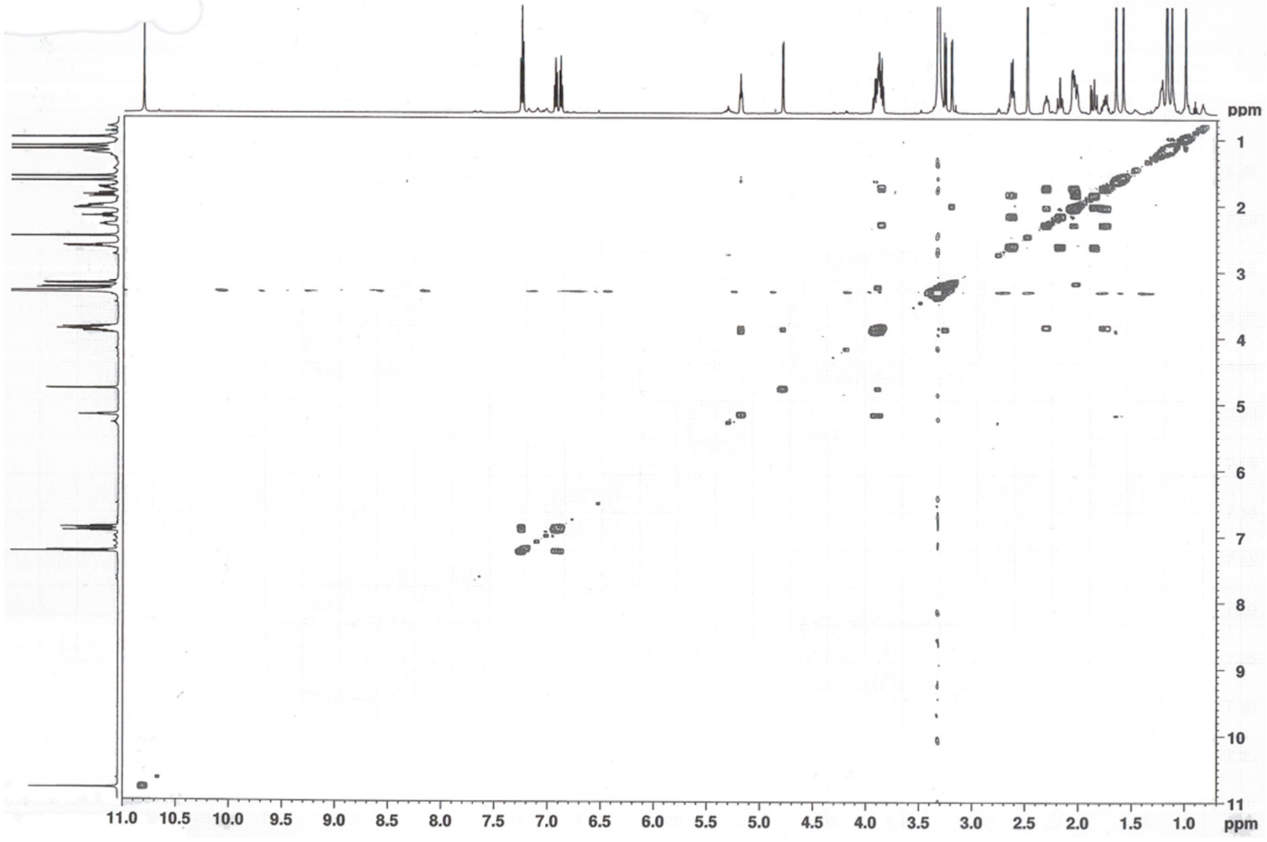

Figure S22. ^{1}H-^{1}H COSY spectrum of terpendole P (**3**) in DMSO- *d*6.

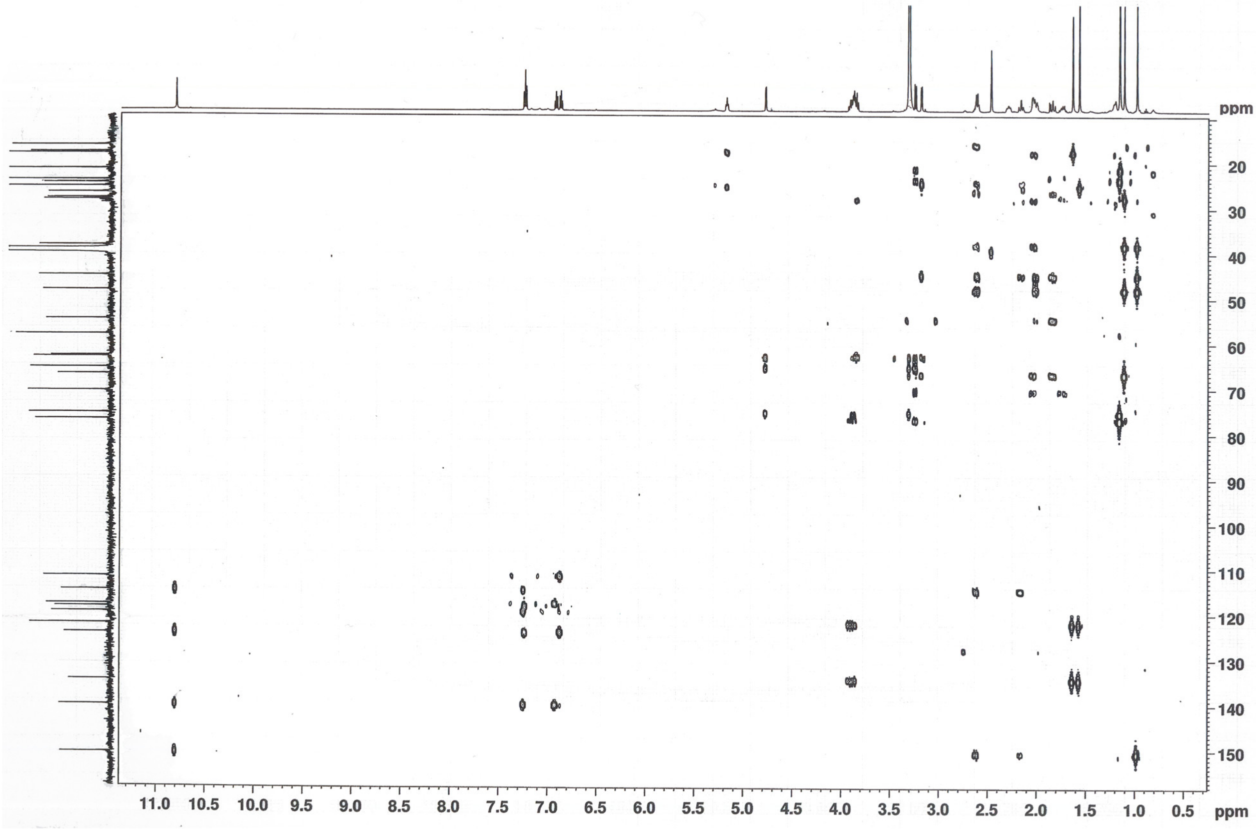

Figure S23. HMBC spectrum of terpendole P (**3**) in DMSO- *d*6.

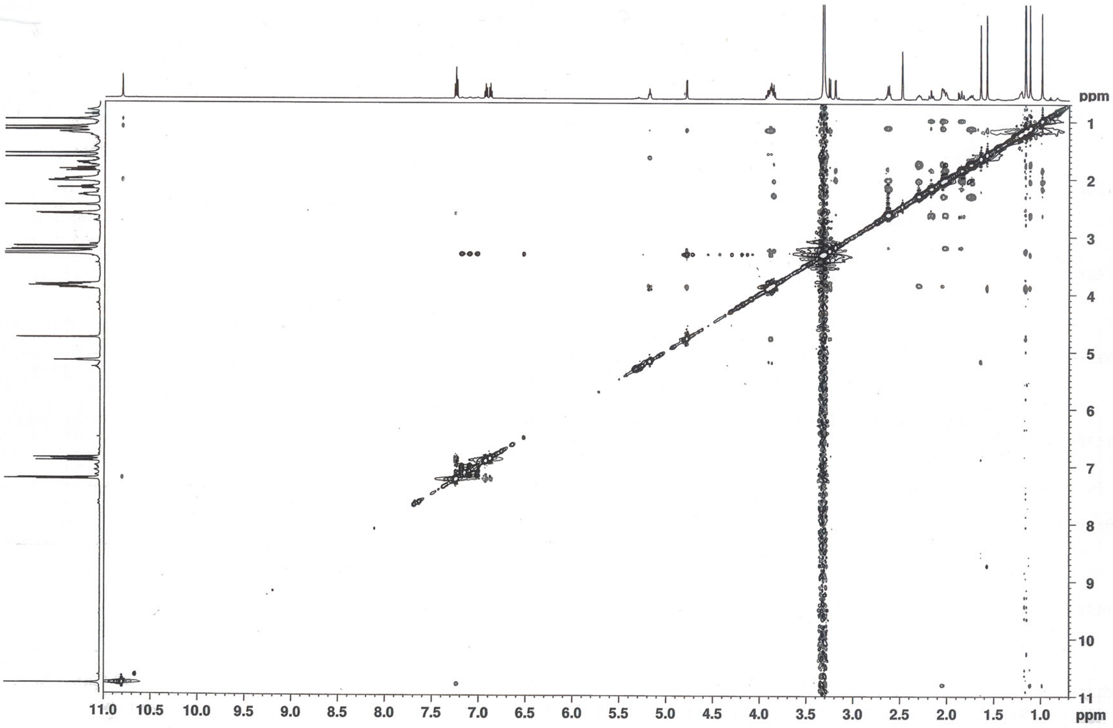

Figure S24. ROESY spectrum of terpendole P (**3**) in DMSO- *d*6.

Supplementary Table 1. ^{1}H and ^{13}C NMR chemical shifts of **2** in $CDCl_3$

Position	Terpendole O (**2**)		
	δ_C ^{a}, type	δ_H ^{b} (multi, J Hz)	HMBC
1-NH	-	7.90 (s)	2, 18, 19, 24
2	151.5, C	-	-
3	50.3, C	-	-
4	42.3, C	-	-
5	27. 4, CH_2	1.34 (t, 6.4)<br>2.70 (br td, 13,4 6.4)	13, 26
6	28, CH_2	2.28 (m)<br>1.78 (m)	4
7	71.5, CH	4.38 (t, 10.0)	9, 11, 12
9	71.18, CH	3.57 (d, 10.0)	7, 27, 28, 29
10	71.12, CH	3.91 (d, 9.6)	27
11	61.1, CH	3.61 (s)	7
12	67.8, C	-	-
13	78, C	-	-
13-OH	-	-	-
14	30.2, CH_2	1.43 (br m)<br>1.56 (br s)	-
15	20.5, CH_2	1.60 (br m)<br>1.90 (br m)	-
16	50.2, CH	2.80 (br m)	-
17	29, CH_2	2.60 (br t, 11.2)<br>2.83 (t, 6.0)	2, 18
18	116.7, C	-	-
19	124.7, C	-	-
20	129, C	-	-
21	119.3, CH	6.86 (d, 6.8)	19, 23
22	120.9, CH	7.02 (t, 7.6)	20, 24
23	109.9, CH	7.19 (d, 7.6)	19, 21
24	139.6, C	-	-
25	15.9, CH_3	1.27 (s)	2, 4, 16
26	18.8, CH_3	1.14 (s)	3, 4, 5, 14, 16
27	74.7, C	-	-
28	16.6, CH_3	1.29 (d, 2.8)	9, 27, 29
29	28.2, CH_3	1.29 (d, 2.8)	9, 27, 28
31	92.6, CH	5.53 (d, 6.8)	10, 27, 34
33	121.9, CH	5.30 (d, 6.8)	35, 36
34	139.6, C	-	-
35	18.6, CH_3	1.74 (d, 0.8)	33, 34, 36
36	25.6, CH_3	1.73 (d, 1.2)	33, 34, 35
37	32.7, CH_2	2.98 (m)<br>3.29 (m)	21, 39
38	64.3, CH	3.09 (dd, 5.2)	19, 20
39	58.7, C	-	-
40	18.9, CH_3	1.42 (s)	38, 39, 41
41	24.9, CH_3	1.34 (s)	38, 39, 40

^{13}C (100 MHz) and 1H (400 MHz) spectra were taken on the NMR system 400 MHz spectrometer (Agilent). Chemical shifts are shown with reference to ^{a}$CDCl_3$ as δ 77.0, ^{b}$CDCl_3$ as δ 7.26. Multiplicity of signals as follows: s = singlet, d = doublets, dd = double doublets, t = triplet, m = multi. Coupling constants (Hz) were determined by the ^{1}H-^{1}H decoupling experiments.
